# Genomic and Transcriptomic Landscapes of MEN1-Wild-Type Low-Grade Metastatic Pancreatic NETs Uncover Key Oncogenic Drivers and Targetable Pathways

**DOI:** 10.64898/2025.12.07.692731

**Published:** 2026-01-12

**Authors:** Md Hafiz Uddin, Zaid Mahdi, Irfana Muqbil, Brendon R. Herring, Bart Rose, Husain Y. Khan, Yiwei Li, Amro Aboukameel, Sahar F. Bannoura, Hugo Jimenez, Allan Johansen, Mohammad Najeeb Al-Hallak, Ibrahim Azar, Amr Mohamed, Tarik Hadid, Nitin Vaishampayan, Yang Shi, Yin Wan, Vy Ong, Gregory Dyson, Rafic Beydoun, Miguel Tobon, Eliza W. Beal, Herbert Chen, Anthony F. Shields, Philip A. Philip, Jennifer Beebe-Dimmer, Ramzi M. Mohammad, Boris C. Pasche, Bassel F. El-Rayes, Asfar S. Azmi

## Abstract

Sporadic pancreatic neuroendocrine tumors (pNETs) with wild type MEN1 represent a major yet largely ignored subset whose biology and metastatic potential remain poorly understood. Because metastasis can occur despite low histologic grade and modest mutational burden, we hypothesized that metastatic competence in MEN1-wild-type pNETs reflects quantitative reinforcement of shared oncogenic pathways rather than distinct mutational processes. We profiled 75 primary low-grade pNETs by whole-exome and RNA sequencing, including 25 percent with lymph node and/or liver metastasis, and integrated genomic and transcriptomic data to connect pathway lesions with expression state. Metastatic tumors showed a slight increase in mutation frequency but conserved base-substitution spectra relative to non-metastatic cases, and adverse clinicopathologic features were enriched in Grade 2 disease. Aggregating alterations to pathways revealed broad convergence on canonical networks, with transcriptomic analyses demonstrating cohort-wide enrichment of Calcium, WNT, and KRAS/PI3K-AKT programs in metastasis. Intersection of significantly mutated genes with differentially expressed genes identified a focused 29-gene overlap, including RYR1 and ZNF273, that marks these convergent axes and distinguishes metastatic from non-metastatic tumors. Gene set enrichment confirmed preferential activation of Calcium, WNT, and PI3K-AKT signaling in metastatic tumors, consistent with a network-intensity model of progression. Finally, upstream-regulator analysis (iPathwayGuide) and gene-centric perturbation mapping (Gene2Drug) nominated candidate targeted and repurposable agents predicted to reverse the metastatic expression phenotype and flagged drugs unlikely to provide benefit, yielding a prioritized, testable therapeutic shortlist which includes fasudil and spaglumic acid. Convergent, domain-specific mutational patterns in highly mutated genes such as ZNF273 and CLCA1 define a molecular signature that could stratify metastatic risk in low-grade pNETs. Collectively, our data reframe metastasis in MEN1-wild-type low-grade pNETs as a property of pathway state rather than mutation quantity and provide a translational blueprint for biomarker-guided therapy development focused on Calcium, WNT, and KRAS/PI3K hubs.

## Full Text Availability

The license terms selected by the author(s) for this preprint version do not permit archiving in PMC. The full text is available from the preprint server.

